# Perception of Sleep Disturbances due to Bedtime Use of Blue Light-Emitting Devices and Its Impact on Habits and Sleep Quality among Young Medical Students

**DOI:** 10.1155/2019/7012350

**Published:** 2019-12-24

**Authors:** Asmaa Jniene, Leila Errguig, Abdelkader Jalil El Hangouche, Hanan Rkain, Souad Aboudrar, Mustapha El Ftouh, Taoufiq Dakka

**Affiliations:** ^1^Exercise Physiology and Autonomic Nervous System Team “EPE-SNA”, Laboratory of Physiology, Faculty of Medicine and Pharmacy, Mohammed V University, Rabat, Morocco; ^2^Department of Pulmonology, Ibn Sina Hospital, Ibn Sina University Hospital Center, Faculty of Medicine and Pharmacy, Mohammed V University, Rabat, Morocco; ^3^Laboratory of Physiology, Faculty of Medicine and Pharmacy, Abdlemalek Essaadi University, Tangier, Morocco

## Abstract

**Introduction:**

The use of blue light-emitting devices (smartphones, tablets, and laptops) at bedtime has negative effects on sleep due to light stimulation and/or problematic excessive use. We aimed to evaluate, among young medical students, if the perception of sleep disturbances due to bedtime use of these devices is consistent with healthier habits and a better sleep quality.

**Materials and methods:**

294 medical students in medicine and pharmacy from the Faculty of Medicine and Pharmacy of Rabat, Morocco, took part in this anonymous and voluntary cross-sectional study and answered an electronic questionnaire. Student and Mann–Whitney *U* tests were used to compare variables between 2 groups based on their perception of sleep disturbances. The level of significance was *p* ≤ 0.05.

**Results:**

286 students (97.3%) used a blue light-emitting smart device at bedtime before sleep, and sleep quality was poor (Pittsburgh Sleep Quality Index, PSQI > 5) in 101 students (35.3%). The perception of sleep disturbances due to this night usage was reported by 188 of them (65.7%). In this group, 154 (81.9%) used their device with all the lights turned off in the room (*p*=0.02), 34 (18.1%) put devices under pillows (*p*=0.04), 114 (60.6%) interrupted sleep to check messages (*p* < 0.001), and the mean duration use of these technologies at bedtime was 2 h ± 23 min per night (*p*=0.02). Also, the mean sleep duration was 6.3 hours ± 1.25 (*p*=0.04), 119 (63.3%) presented fatigue on waking more than one time per week (*p*=0.04), and 76 (40.4%) presented poor sleep quality (75.2% of the students with PSQI > 5) (*p*=0.005).

**Conclusions:**

Despite the perception of sleep disturbances due to bedtime use of blue light-emitting devices, unhealthy sleep habits tend to be frequent in young medical students and worrying because it is associated to significant poor sleep quality.

## 1. Introduction

The use and accessibility of blue light-emitting devices such as smartphones, tablets, and laptops has increased widely in the last decade [1]. These technologies have had a radical effect on the social transformation process with an impact on daily routines and habits and had become an integral part of human life [2, 3]. Used wisely, they are clearly useful, practical, and constitute an excellent mean of communication, production, and entertainment. However, these devices, that have become more lightweight and portable making them easier to use, have become such indispensable and a huge part of our daily lives including at bedtime.

It is assumed that impaired sleep leads to many serious consequences in health as overweight/obesity or mood disorders and increased accidents [1, 2, 4–13]. Appropriate behavior and environment are thus critical to achieve adequate quality and quantity of sleep. In this context, several studies showed the negative effects of bedtime blue light-emitting-device use on sleep [1, 4, 6, 7, 14–18]. On one hand, there is a cognitive stimulation and related sleep disturbances due to the brightness of the short blue wavelengths light emitted by these devices that disrupt the circadian clock [1, 4, 6, 7, 14–16].

On the other hand, there is an emergent problematic and worrying excessive use of these new technologies in people of all ages reported by several studies [9, 18–28]. As we are all conscious of bedtime, wake time and the subjective quality of sleep, our appreciation of the importance of the sleep-wake cycle should be further enhanced when confronted with the consequences of its disruption [29]. In this context, data assessing the perception of sleep disturbances due to the night use of smart devices and its impact on sleep habits are limited. A study realized on university students and based on Sleep Hygiene Practices Scales and the Pittsburgh Sleep Quality Index (PSQI) suggested that knowing about proper habits in general does not necessarily influence sleep quality, whereas practicing proper habits is strongly related to good overall sleep quality [30]. Another study realized on primary and secondary school students investigated the prevalence and patterns of the specific smart device activities and purposes and perceived outcomes (including the perception of sleep deprivation). The results showed that despite the one-week prevalence of perceived sleep deprivation (nearly 50%), adolescents spent significantly more time per day on messaging, browsing information, and gaming than those who did not perceive these outcomes [31].

To the best of our knowledge, there is no previous reports assessing the perception of sleep disturbances due to bedtime use of blue light-emitting devices in young adults and its impact on sleep hygiene and sleep quality. In this study, we hypothesized that students who perceived bedtime blue light-emitting-device use as detrimental to sleep quantity and quality would have better sleep quality than those who did not because they would adopt healthier sleep habits (including less bedtime blue light-emitting-device use).

We conducted our study among young undergraduate students in the Faculty of Medicine and Pharmacy in Rabat, Morocco, given that sleep plays an essential role in restoring energies expended during the day, in memorization, concentration, and learning processes [32, 33] and given that university students are known in general for their variable sleep schedules [30, 34, 35].

## 2. Materials and Methods

### 2.1. Sampling and Study Population

This is an anonymous, voluntary, prospective, and observational cross-sectional study based on an online questionnaire. The Ethical Committee of the Faculty of Medicine and Pharmacy, Mohammed V University, Rabat, Morocco, approved the study protocol in accordance with the Declaration of Helsinki (1964).

The questionnaire was developed via the Google Forms platform and posted in groups created on a social network, composed of students in medicine and pharmacy from the Faculty of Medicine and Pharmacy of Rabat, Morocco. These groups enable the students to communicate and to share information, comments, messages, videos, and images.

The notice of information was detailed in the home page. By clicking the next button that appeared in the bottom of the page and as explained in the notice of information, participants declared they have read the full information note and consented to take part to this study. The Ethical Committee approved this procedure.

294 students were included in the study. The sample size was determined first by calculating the sample size for infinite population (S) based on formula: *S* = *Z*^2^ × *p* × (1 − *p*)/*M*^2^ = 384.16, where *Z* = *Z*-score determined based on the confidence level considered in this study at 95% which refers to a *Z*-score at 1.960, *p* = population proportion assumed to be 50%, and *M* = margin of error considered 0.5 in this study. The sample size was then calculated by adjusting the sample size for infinite population to target population by the following formula = (*S*)/1 + [(*S* − 1)/population], where target population was based on the assumption of 1240 students for this study.

The included students owned a blue light-emitting smart device (a smartphone and/or a tablet and/or a laptop), did not realize yet night shifts (students from the first to the fifth year of Medicine and students in Pharmacy), were registered in groups created on a social network as mentioned above, and were not followed for a psychiatric nor a chronic disease or presented an acute illness during the month preceding the questionnaire response (example: a respiratory tract infection, a urinary infection…) or any personal issue that may interfere with sleep (example: a separation, an accident…).

### 2.2. Measurements

Students responded to an online questionnaire that consisted of two parts with an average duration to answer all the questions of 10 minutes. A preliminary interview with the students' representatives was carried out in order to set the average duration not to be exceeded for completing the questionnaire. Lengthy surveys are likely to overburden participants and increase inaccuracies if participants rush or omit information to finish. Factors associated with response burden are questionnaire length in particular, cognitive ability to complete the survey, and type of questionnaire interface [36].

All the questions included in the questionnaire were made required while setting the form in order to have no missing values.

The first part of the questionnaire gathered sociodemographic information (including gender, age, height, weight, and study level), data on the bedtime use of the devices in the last month prior to the response (April 2018 for this study), and students' perception of sleep disturbances as a consequence (i.e., do you think and perceive that using blue light-emitting devices at bedtime leads to sleep disturbances in terms of quantity and/or quality?).

Data on the blue light-emitting devices bedtime habits included mainly the automatic or manual brightness adjustment, lights off or on in the bedroom, the main reason for bedtime use (reading use for academic program or leisure use including social networking sites, instant messaging, websites surfing, games…), the mean duration, devices switched off or not before sleep, smartphones or tablets put under pillow while sleep, interruption of sleep to check messages, and also daytime symptoms that may appear upon awakening due to impaired sleep, i.e., fatigue on waking, headaches, irritability.

The screens of TVs, although they display a similar intensity of blue light, were excluded from this study since the interaction with the device is less active and the eye, farther away, receives less blue light [37].

The second part of the questionnaire comprised the Pittsburgh Sleep Quality Index (PSQI) (translated in French [38]) which is a self-report questionnaire that assesses sleep quality over the previous month. We assessed the global PSQI score and the scores of the 7 components (C1 to C7), which were determined by using a four-grade system (0, 1, 2, and 3). C1 = subjective sleep quality was assigned a score of 0–3 points (0 = very good sleep quality; 1 = fairly good sleep quality; 2 = fairly bad sleep quality; and 3 = very bad sleep quality); C2 = sleep latency (time to fall asleep), 0 ≤ 15 min, 1 = 16–30 min, 2 = 31–60 min, 3 ≥ 60 min; C3 = sleep duration, 0 = sleep duration > 7 hours; 1 = 6-7 hours; 2 = 5-6 hours; and 3 ≤ 5 hours; C4 = habitual sleep efficiency (ratio of hours asleep to hours in bed) 0 = 85%, 1 = 75–84%, 2 = 65–74%, 3 ≤ 65%; C5 = sleep disturbance (trouble sleeping for some reason and the frequency), 1 = Score: 0, 2 = Score: 1, 3 = Score: 2, 4 = Score : 3; C6 = use of sleeping medication, 1 = not during the past month, 2 = less than once a week, 3 = once or twice a week, 4 = Three or more times a week and C7 = daytime dysfunction, 1 = Score: 0, 2 = Score : 1, 3 = Score : 2, 4 = Score: 3.

The global PSQI score was calculated by totaling the seven component scores, providing an overall score ranging from 0 to 21. A global PSQI score greater than 5 yielded a diagnostic sensitivity of 89.6% and specificity of 86.5% in distinguishing good and poor sleepers [39].

### 2.3. Statistical Analysis

Statistical Analysis System IBM SPSS Statistics V20.0.0 was used to analyze survey data. 2 groups were compared based on the perception of sleep disturbances as a consequence of blue light-emitting devices bedtime use. Quantitative values were expressed as mean and standard deviation (SD) or median (interquartile lower, upper). Quantitative values were analyzed by the Student test when the measures were normally distributed or by nonparametric test (Mann–Whitney *U*) when the measures were not normally distributed (Kolmogorov–Smirnov test was used to test normality). Qualitative values were expressed in number and percentage of all students and analyzed by the chi^2^ test. *p* values of less than 0.05 were considered statistically significant.

## 3. Results

294 students took part in this study (61.2% of women), their mean age was 20.6 ± 1.8 years. 230 (78.2%) studied medicine and 64 (21.8%) pharmacy.

There was no significative statistical difference between males and females for any variable, neither for the year or field of studies nor the Body Mass Index.

### 3.1. Blue-Light-Device Bedtime Habits and Sleep Quality for all Students

**Figure 1:**
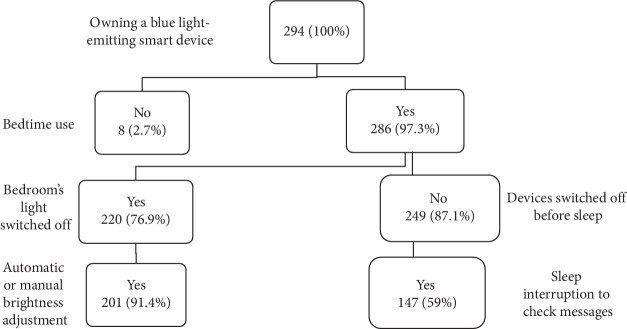
Blue-light-device bedtime habits for all students.

Figure 1 shows that most students (97.3%) used a blue light-emitting smart device at bedtime. Among this population, 76.9% used them with the lights off in the bedroom. Furthermore, 249 (87.1%) students did not switch off their smartphones or tablets before sleeping from whom 48 (19.2%) put it under the pillow (respectively, 45.5% and 10.4% of women), and 147 (59%) interrupted sleep to check messages (31.7% of women). The main reason for bedtime use was leisure in 217 (75.9%) students and reading in 72 (25.2%) students (respectively, 44.6% and 13.6% of women). The mean duration of screen use at bedtime immediately before sleep was 1 h 50 ± 25 min per night (1 h 43 ± 24 for men versus 1 h 55 ± 29 for women).

Fatigue upon waking, impression of not having slept enough, irritability, and headaches are symptoms that may appear upon awakening due to impaired sleep. Their prevalence was, respectively, 85.7%, 87.1%, 68.9%, and 57.7%. We note that 48.6% of woman reported fatigue upon waking.

Across all students, the mean sleep onset latency was 18.1 min ± 14.4, the mean average daily sleep time was 6.52 hours ± 1.29, and poor sleep quality (PSQI score > 5) was reported by one-third of participants (35.3% from whom 20.6% of woman).

### 3.2. Blue-Light-Device Bedtime Habits and Sleep Quality according to Perception of Sleep Disturbances as a Consequence

When the students were asked if they think and perceive that using blue light-emitting devices at bedtime leads to sleep disturbances in quality and/or quantity, 188 (65.7%) answered yes. In this population, sleep habits and symptoms that may appear upon awakening due to impaired sleep are represented in Table 1 and sleep quality in Table 2.

Table 1 shows that, in the group of students who perceived sleep disturbances due to bedtime use of blue light-emitting devices and compared to those that did not perceive these disturbances, 154 (53.8%) switched off the light while using their devices (*p*=0.02), 34 (11.9%) put their smartphones or tablets under the pillow while sleep (*p*=0.04), and 114 (39.9%) interrupted their sleep to check messages (*p* < 0.001). Moreover, 119 students (41.6%) reported more fatigue upon wakening (*p*=0.04).

The main reason for bedtime use was leisure in 172 (91.5%) students who perceived sleep disturbances compared to those who did not (*p* < 0.001). The mean duration of screen use at bedtime immediately before sleep was 2 h ± 23 min per night in students who perceived sleep disturbances versus 1 h ± 19 min (*p*=0.02) in those who did not.

Table 2 displays the results of the PSQI global score and by components (C1–C7). The mean global PSQI score was 5.62 ± 2.91, and 35.3% of the students had a score >5, indicating poor sleep quality. Regarding the components of the PSQI, the score was highest for C1 (subjective sleep quality), followed by C3 (sleep duration), then C5 (sleep disturbances). The mean sleep duration was 6.3 hours ± 1.25 in students that perceived sleep disturbances due to bedtime use of blue light-emitting devices versus 7.21 hours ± 1.23 (*p*=0.04), the mean sleep efficiency was 87.7% ± 13.7, and the median sleep latency was 17.5 minutes (9.5, 26.2).

Among the students with poor quality sleep, 76 students (40.4%) reported a perception of sleep disturbances as a consequence of bedtime use of blue light-emitting devices (75.2% from PSQI > 5, *p* < 0.005). In this population, the mean score of C1 (subjective sleep quality), C3 (sleep duration), and C7 (daytime dysfunction) were statistically higher (*p*=0.01, *p*=0.01, *p*=0.005, respectively).

## 4. Discussion

Our results showed that most of the students (97.3%) used blue light-emitting devices at bedtime before sleep of whom 35.3% presented a poor quality of sleep (PSQI > 5). The perception of sleep disturbances due to this night usage was reported by 188 of them (65.7%). Among them and compared to those who did not perceive sleep disturbances, unhealthy sleep habits were significantly more frequent (including a significantly higher mean duration use of the devices per night) and associated to significant poorer sleep quality. These findings were the opposite of our expectation as we hypothesized that students who perceived sleep disturbances due to bedtime blue light-emitting-device use would have better sleep quality than those who did not because they would adopt healthier sleep habits including less bedtime blue light-emitting device use.

Our study showed first the extent to which students used blue light-emitting devices at bedtime (97.3% of all students). These findings support several prior studies showing that technology use near bedtime is extremely prevalent [35, 40]. In general, with the huge progress of technology from which interactivity, touchscreen technology, and the growing number of software and internet-based applications' develop, portable devices have become essential devices thoroughly integrated into our daily lives. Settling down with this technology in bed for instance has become a nightly routine for many adults [41]. Contradictorily, sleep hygiene necessities an appropriate environment including a dark and quiet environment. In the present study, sleep hygiene seemed to be poor in general: the mean duration of screen use at bedtime immediately before sleep was 1 h 50 ± 25 min per night, 76.9% of the students used devices with the lights off in the bedroom, 87.1% did not switched off their smartphones or tablets before sleeping, and 59% interrupted their sleep to check messages (Figure 1). Researches showed that most young adults (57 to 60%) leave their phone on during sleep [35, 41], with only 33% turning it to silent or vibrate modes [35]. A study showed the most obvious aspect connecting mobile phone use with sleep disturbances was being awakened at night by phone calls or messages [42].

The 2011 Sleep in America Poll addressed technology available in the bedroom. “Generation Y'ers,” adults aged 19–29 years old are heavy users of technology prior to bed with 67% using cell phones. The majority (51%) report rarely getting a good night's sleep and often wake unrefreshed [40]. Indeed, fatigue upon waking, impression of not having slept enough, irritability, and headaches are symptoms that may appear upon awakening due to impaired sleep. Their prevalence was high in our study and found in, respectively, 85.7%, 87.1%, 68.9%, and 57.7%.

Besides these findings, the mean average daily sleep time was low 6.52 hours ± 1.29 and poor sleep quality (PSQI score > 5) was reported by one-third of participants (35.3%). Through the past 20 years, researchers have identified a reduction in the average number of hours of sleep among college students [43] and poor sleep quality found up to 50% [44] which negatively impacts learning and memory processes [32, 33, 45]. We note also that data suggest a potential causal relationship between poor sleep and greater rates of weight gain [11]. In the present study, there was no significative statistical difference between sleep quality and the Body Mass Index and future research is needed in our context with a greater number of participants.

Several studies showed the negative effects of bedtime blue light-emitting-device use on sleep due to its association to cognitive stimulation [1, 4, 6, 7, 14–18] and/or to problematic excessive use [9, 18–28].

The cognitive stimulation is a transversal issue in all the students in our study. Multiple studies and concerns have emerged over the past fifteen years and showed a cognitive stimulation and related sleep disturbances leading to impaired sleep by using blue light-emitting devices in the evening close or at bedtime [1, 2, 4, 8, 46]. Studies have shown that even low intensity light can act on the circadian clock [2, 47]. For instance, we found that 139 (48.6%) students despite using the automatic or the manual brightness adjustment reported a perception of a negative impact on sleep (Table 1).

In this study, we hypothesized that the use of blue light-emitting devices at bedtime may represent a disturbance in the timing of sleep cycles and the perception of its negative impact should be consistent with a healthier sleep habits, including a less use of the blue light-emitting devices at bedtime and would lead to a better sleep quality. A study realized in 2017 investigated the prevalence and patterns of smart device activities and purposes and frequencies of perceived outcomes (including the perception of sleep deprivation) related to smart device use and the differences in patterns of smart device activities within a specific timeframe between 2 groups who did and did not perceive the outcomes. This study was realized on primary and secondary school students aged between 10 and 19 years [31]. The one-week prevalence of perceived sleep deprivation was nearly 50%. Despite this perception, adolescents spent significantly more time per day on messaging, browsing information, and gaming than those who did not perceive these outcomes [31]. Our results showed a higher rate of sleep disturbances perception due to this night usage in 188 students (65.7%). Despite this finding, in this group, sleep habits seemed to be also poorer compared to those who didn't report this perception. Indeed, the mean duration of screen use at bedtime immediately before sleep was 2 h ± 23 min per night, and the main reason for the use was leisure. Also, 154 students among those who perceived sleep disturbances (81.9%) switched off the ambient light while using their devices (*p* = 0.02), 34 (18.1%) put their smartphones or tablets under the pillow while sleep (*p* = 0.04), and 114 (60.6%) interrupted their sleep to check messages (<0.001) (Table 1). Moreover, 119 students (63.3%) reported more fatigue upon wakening (*p* = 0.04), and poor sleep quality was highly prevalent (75.2% of the students with PSQI global score >5) (*p* < 0.005) (respectively, Tables 1 and 2). In this population, the scores of subjective sleep quality, sleep duration, and daytime dysfunction were statistically higher (*p* = 0.01, *p* = 0.01, *p* = 0.005, respectively) (Table 2). There was no statistical difference in terms of sleep latency or habitual sleep efficiency. This is probably due to an important sleep propensity in these students because of the sleep deficit.

These general findings were the opposite of our expectations. In our study, using blue light-emitting devices despite the perception of its negative impact on sleep may be also attributed in addition to light stimulation to a problematic excessive use to a certain extent. Problematic or excessive mobile phone use refers to an individual's inability to control their usage of their mobile phone leading to adverse consequences in their everyday life such as sleep disturbances [19]. This problematic use is also inferred in physically hazardous situations while driving or crossing the road for example. Recent several studies reported this problematic excessive use related especially to smartphones, online social networking, and internet use in general [20–22, 28, 48–51] and to be accompanied by symptoms found in addictive/substance use disorders, including dependence and withdrawal when not using one's smartphone [18]. These studies were based on scales as the internet or smartphone addiction scales. In our study, we were directly interested by the perception and awareness of the students' perception of sleep disturbances due to bedtime use of blue light-emitting devices. In this group and despite their perception, they reported a use mainly for leisure including social networking sites, instant messaging, websites surfing, games… These sites use compulsive and captivating interfaces and rely on a discipline born in the last decade: Captology (acronym for Computer As Persuasive Technology) [52, 53]. On the contrary, in the group who did not perceive this outcome, the use of the devices was mainly for academic reading with lower mean duration of screen use at bedtime immediately before sleep that is 1 h ± 19 min per night versus 2 h ± 23 min in those who perceived the negative outcomes (*p*=0.02).

Several studies found no gender differences as concern the problematic excessive use [54, 55]; nevertheless, psychobehavioral factors were differently associated: game apps use, anxiety, and poor sleep quality for males versus multimedia applications' use, social networking services' use, depression, anxiety, and poor sleep quality for females [55]. In this context, we note that sleep disturbance is an important but underrecognized mechanism in the multifactorial cause and maintenance of the mood disorders as depression and anxiety [56]. In our study, no significant statistical gender differences were found (blue-light-device bedtime habits and principal use, sleep quality, perception of sleep disturbances, daytime symptoms that may appear upon awakening due to impaired sleep).

The main limitations of our study are represented by the answers collected that are not necessarily representative of all university students despite the large diffusion of the study. Also, the information were collected by a questionnaire including the PSQI. This questionnaire suffers from the same problems as other self-report inventories in that scores can be easily exaggerated or minimized by the person completing them and most of information are subjective symptom-reports. On the other hand, like all questionnaires, the way the instrument is administered can influence the final score (online questionnaire for this study).

A further limitation is the difficultly in establishing causality. Longitudinal studies are thus required to clarify the cause-and-effect relationships. Indeed, sleep problems are likely multicausal, comprising a range of physical, mental, social, and behavioral determinants. Variables that can cause irregular sleep patterns such as napping, caffeine and/or energy drinks consumption, lifestyles and diseases [57], circadian type (morningness), and sleep timing regularity were not assessed in the students. Also, anxiety and/or depression is caused by personal, social, or educational stressors. Indeed, the individual type of stress response could occur through behavioral changes including sleep [58, 59]. Other psychological measures also need to be included such as the smartphone and/or internet addiction scale as we suggested the problematic excessive use of these new technologies as well as a face to face investigation or the use of a diary. More longitudinal researches considering these limitations are required to reach more definitive conclusions.

## 5. Conclusion

Our study has shown a high prevalence in using blue light-emitting devices at bedtime as well as worrying and unhealthy habits associated to poor sleep quality and affecting daytime functioning among young medical students of both genders. Despite the perception of sleep disturbances due to this bedtime use, we significantly found an association to poorer sleep habits, poorer sleep quality, more fatigue, and a use for leisure mainly. These general findings were the opposite of our expectations: using blue light-emitting devices despite the perception of their negative impact on sleep may be attributed in addition to light stimulation, to a problematic excessive use to a certain extent.

Minimizing devices access at bedtime is warranted and future research is needed to evaluate the effectiveness of interventions for improving this condition and the impact on sleep hygiene as well as outcomes.

## Figures and Tables

**Table 1:** Blue-light-device bedtime habits and symptoms that may appear upon awakening due to impaired sleep according to perception of sleep disturbances as a consequence.

	Sleep disturbances perception as a consequence of bedtime use of blue light-emitting devices		
	Yes (*N*: 188)	No (*N*: 98)	*p*
Lights of bedroom switched off	154 (53.8%)	66 (23.1%)	0.02
Automatic or manual brightness adjustment activated	139 (48.6%)	62 (21.7)	0.3
Smartphones or tablets are not switched off before sleep	161 (56.3%)	88 (30.8%)	0.1
Smartphones or tablets put under pillow while sleep	34 (11.9%)	14 (4.9%)	0.04
Interruption of sleep to check messages	114 (39.9%)	33 (11.5%)	<0.001
Fatigue on waking > 1 time per week	119 (41.6%)	50 (17.5%)	0.04
Headaches > 1 time per week	50 (17.5%)	22 (7.7%)	0.08
Irritability > 1 time per week	72 (25.2%)	31 (10.8%)	0.4

Results are expressed as number (percentage of all students).

**Table 2:** The PSQI global score and by components (C1–C7) according to sleep disturbances perception as a consequence of bedtime use of blue light-emitting devices.

PSQI	All (N: 286)	Sleep disturbances perception as a consequence of bedtime use of blue light-emitting devices		
		Yes (*N*: 188)	No (*N*: 98)	*p*
PSQI global score >5	101 (35.3%).	76 (26.6%)	25 (8.7%)	0.005
PSQI mean score	5.62 ± 2.91	6.45 ± 3.3	5.41 ± 3.11	0.005
Component 1: subjective sleep quality	1.70 ± 0.98	1.89 ± 1.12	1.54 ± 0.62	0.01
Component 2: sleep latency	0.76 ± 0.89	0.75 ± 0.80	0.76 ± 0.69	0.2
Component 3: sleep duration	1.45 ± 0.84	1.67 ± 0.62	1.34 ± 0.79	0.01
Component 4: habitual sleep efficiency	0.72 ± 0.93	0.74 ± 0.98	0.67 ± 0.92	0.2
Component 5: sleep disturbances	0.94 ± 0.66	0.99 ± 0.64	0.91 ± 0.65	0.2
Component 6: use of sleeping medication	0.18 ± 0.33	0.15 ± 0.30	0.12 ± 0.36	0.6
Component 7: daytime dysfunction	0.66 ± 0.79	0.69 ± 0.85	0.68 ± 0.76	0.005
Sleep duration (hours)	6.52 ± 1.31	6.3 ± 1.25	7.21 ± 1.23	0.04
Sleep efficiency (%)	87.4 ± 13.3	87.7 ± 13.7	89.8 ± 12.6	0.1
Sleep latency (minutes)	17.5 (9.5, 26.2)	16.1 (11.6, 27.3)	19.2 (8.9, 24.6)	0.07

Quantitative values are expressed as means ± SD or median (interquartile lower, upper). Qualitative values are expressed as number (percentage of all students).

## Data Availability

The data used to support the findings of this study are available from the corresponding author upon request.
